# Large ischemic core defined by visually assessed ASPECTS predicts functional outcomes comparably accurate to automated CT perfusion in the 6–24 h window

**DOI:** 10.1177/23969873241286691

**Published:** 2024-10-07

**Authors:** Tolga D Dittrich, Anh Nguyen, Peter B Sporns, Anna M Toebak, Lilian F Kriemler, Salome Rudin, Annaelle Zietz, Benjamin Wagner, Filip Barinka, Martin Hänsel, Henrik Gensicke, Raoul Sutter, Christian H Nickel, Mira Katan, Nils Peters, Lars Michels, Zsolt Kulcsár, Grzegorz M Karwacki, Marco Pileggi, Carlo Cereda, Susanne Wegener, Leo H Bonati, Marios Psychogios, Gian Marco De Marchis

**Affiliations:** 1 Department of Neurology and Stroke Center, University Hospital Basel and University of Basel, Basel, Switzerland; 2 Department of Neurology and Stroke Center, Cantonal Hospital St. Gallen, St. Gallen, Switzerland; 3 Department of Clinical Research, University of Basel, Basel, Switzerland; 4 Department of Neuroradiology, University Hospital Basel, Basel, Switzerland; 5 Department of Intensive Care Medicine, University Hospital Zurich and University of Zurich, Zurich, Switzerland; 6 Department of Neurology and Stroke Center, Hirslanden Hospital Zurich, Zurich, Switzerland; 7 Department of Neurology, University Hospital and University of Zurich, Zurich, Switzerland; 8 Neurology and Neurorehabilitation, University Department of Geriatric Medicine Felix Platter, Basel, Switzerland; 9 Medical Faculty, University of Basel, Basel, Switzerland; 10 Department of Intensive Care Medicine, University Hospital Basel and University of Basel, Basel, Switzerland; 11 Emergency Department, University Hospital Basel and University of Basel, Basel, Switzerland; 12 Department of Neuroradiology, University Hospital Zurich, Zurich, Switzerland; 13 Neuroscience Center Zurich, University of Zurich and Swiss Federal Institute of Technology Zurich, Zurich, Switzerland; 14 Department of Radiology and Nuclear Medicine, Cantonal Hospital of Lucerne, Lucerne, Switzerland; 15 Department of Neuroradiology, EOC Neurocenter of Southern Switzerland, Lugano, Switzerland; 16 Department of Neurology and Stroke Center, EOC Neurocenter of Southern Switzerland, Lugano, Switzerland; 17 Rheinfelden Rehabilitation Clinic, Rheinfelden, Switzerland

**Keywords:** Ischemic stroke, CT perfusion, ASPECTS, endovascular treatment

## Abstract

**Introduction:**

Automated CT perfusion (aCTP) is commonly used to select patients with anterior circulation large vessel occlusion (aLVO) for endovascular treatment (EVT). The equivalence of visually assessed Non-contrast CT Alberta Stroke Program Early CT Scores (ASPECTS) and aCTP based selection in predicting favorable functional outcomes remains uncertain.

**Patients and methods:**

Retrospective multicenter study of adult aLVO patients from the Swiss Stroke Registry (2014–2021) treated with EVT or best medical treatment 6–24 h after stroke onset. We assessed ASPECTS on non-contrast CT visually and ischemic core volumes on aCTP, defining ASPECTS 0–5 and aCTP CBF < 30% volumes ⩾50 mL as large ischemic cores. We used logistic regression to explore the association between CT modalities and favorable functional outcomes (modified Rankin Scale [mRS] score shift toward lower categories) at 3 months. Receiver operating characteristic (ROC) curve analysis compared the predictive accuracy of visually assessed ASPECTS and aCTP ischemic core for favorable outcomes (mRS 0–2) at 3 months.

**Results:**

Of 210 patients, 11.4% had ASPECTS 0–5, and 12.9% aCTP core volumes ⩾50 mL. Within the same model, ASPECTS but not aCTP core volumes were associated with favorable outcomes (ASPECTS: acOR 1.85, 95%CI 1.27–2.70, *p* = 0.001). The ROC curve analyses showed comparable diagnostic accuracy in predicting favorable functional outcomes (mRS 0–2) at 3 months (ROC areas: ASPECTS 0.80 [95%CI 0.74–0.86] vs aCTP core 0.79 [95%CI 0.72–0.85]).

**Discussion and conclusion:**

In patients with aLVO, visually assessed ASPECTS showed at least comparable accuracy to automatically generated CTP core volumes in predicting functional outcomes at 3 months.

## Graphical abstract

**Graphical abstract:**
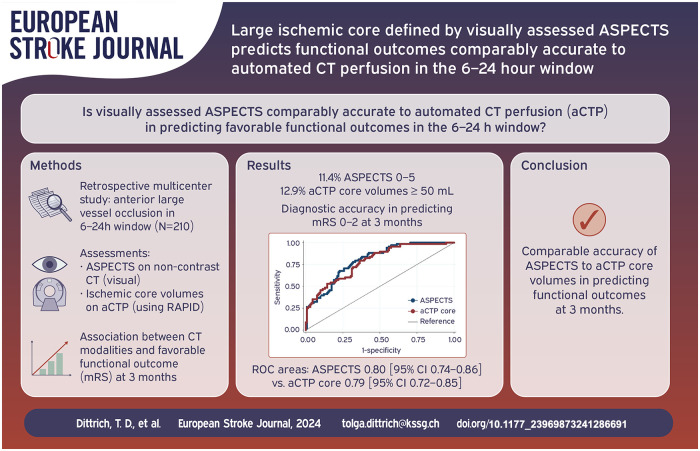

## Introduction

Recent clinical trials demonstrated the benefit of endovascular treatment (EVT) within 24 h for patients with anterior circulation large vessel occlusion (aLVO) and large ischemic core.^1–3^ In SELECT2 and ANGEL-ASPECT, ischemic core size was assessed primarily on non-contrast computed tomography (NCCT) or automated CT perfusion (aCTP; software used in both trials: RAPID by iSchemaView), whereas in RESCUE-JAPAN mostly on MRI.^1–3^

In both SELECT2 and ANGEL-ASPECT, if ASPECTS ranged between 3 and 5 points, aCTP was not needed for patient selection – a common scenario observed in 82% of SELECT2 patients and 86% of ANGEL-ASPECT patients.^1,2^

Compared to aCTP evaluation, NCCT is faster, less prone to motion artifacts and with lower radiation dose delivered.^4,5^ A recent retrospective analysis of an international stroke registry compared the predictive value of visually assessed ASPECTS with aCTP in patients who all underwent EVT.^6^ The authors demonstrated that aCTP core volumes ⩾70 mL were more predictive of poor outcome (defined as modified Rankin Scale [mRS] score 5–6) than ASPECTS ⩽ 5 in EVT patients, especially within 6 h of stroke onset.

Our study aimed to examine the predictive value of visually assessed ASPECTS to aCTP ischemic core volumes using RAPID in a multicenter cohort of aLVO patients admitted within 6–24 h regarding favorable functional outcomes at 3 months using the mRS score.

## Patients and methods

### Study design and data collection

**Figure 1.**
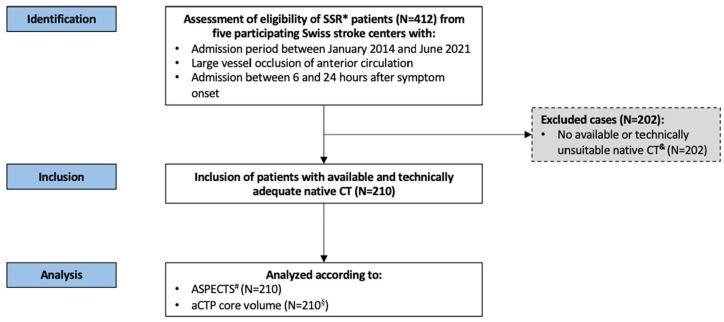
Study flow chart. *Swiss Stroke Registry. ^&^Computed tomography of the head. ^#^Alberta Stroke Program Early CT Score (0–10 points, higher scores indicate less severe early ischemic injury). ^§^Automated computed tomography perfusion ischemic core volume (determined using automated software [RAPID, iSchemaView Inc, Menlo Park, CA]).

We used data from the retrospective multicenter Swiss RESCUE cohort,^7^ based on Swiss Stroke Registry data. Clinical data were collected from the Swiss Stroke Registry from five participating Swiss stroke centers between January 2014 and June 2021. Baseline imaging parameters (ASPECTS, leptomeningeal collateral status, and perfusion imaging parameters) and treatment information (occlusion site and recanalization status [only for EVT patients]) were complemented. Only patients with available ASPECTS were considered (Figure 1). The responsible ethics committee approved the study (ID 2020-00552).

### ASPECTS assessment and functional outcome

ASPECTS assessment was performed visually on NCCT by two neuroradiologists (AN, PBS) blinded to medical history and functional outcomes (mRS score at 3 months with favorable functional outcome defined as a shift toward lower categories). ASPECTS ranges from 0 to 10 points, with lower scores indicating greater ischemic injury.^8^ For baseline comparisons and further analyses, ASPECTS was utilized in both continuous and dichotomized forms (low [0–5 points] vs high [6–10 points]).

### Ischemic core volumes assessment on automated CT perfusion

As previously described, ischemic core volumes on aCTP were assessed using automated software (RAPID, iSchemaView Inc, Menlo Park, CA).^9^ The ischemic core volume was defined as the brain territory with CBF below 30% compared to the contralateral hemisphere.^10^ For the analyses, we used continuous and dichotomized ischemic core volumes (large [⩾50 mL] vs small [<50 mL]; as in the recent SELECT2 trial^2^]).

### Statistical analysis

Categorical and continuous variables are presented as medians with interquartile ranges (IQR), dichotomous variables as numbers with percentages. Group comparisons were conducted using the Wilcoxon rank-sum and Pearson’s chi-square tests. Only individuals with a complete set of covariables were included (Supplemental Table 1).

First, univariable ordinal logistic regression was used to identify predictors of favorable functional outcome at 3 months. Second, multivariable ordinal logistic regression models were fitted, including variables with *p*-values <0.05 from the univariable analyses and variables reported in the literature as predictive of outcome.^11,12^ Two separate models were fitted to examine the association between ASPECTS and aCTP core volumes with 3 months mRS, adjusting for age, sex, occlusion site (internal carotid artery, M1 or M2 segment of the middle cerebral artery), prior mRS score (i.e., before index event), prior ischemic stroke, intravenous thrombolysis, and performance of EVT.

We combined both ischemic core parameters (ASPECTS, aCTP) within one model (adjusting for the same covariables as in the previous models) to compare their predictive utility, considering the different approach of the two parameters to the same concept (i.e., early ischemic damage). We performed the analyses with the dichotomized (high [>5 points] vs low [⩽5 points]) and continuous ASPECTS. The same was done for the aCTP core volume (dichotomized: small [<50 mL] vs large [⩾50 mL] and continuous). Coefficient standardization using z-transformations was performed to promote comparability for the continuous ischemic injury parameters in the combined models. In case of a correlation between ASPECTS and aCTP without evidence of multicollinearity, the results of the combined model were defined to be considered robust since the collective information then does not appear to overlap significantly (with the inclusion of other control variables that help explain variation in the outcome variable).

As a final step, we additionally performed receiver operating characteristic (ROC) curve analysis to assess and compare the predictive accuracy of visually assessed ASPECTS and aCTP ischemic core for outcome prediction (favorable outcome at 3 months, defined as a mRS of 0–2).

Statistical analyses were conducted with STATA version 17.0 (StataCorp LLC, College Station, TX). *p* Values < 0.05 were considered significant. We report 95% confidence intervals.

## Results

### Patient characteristics according to ASPECTS subgroup

Of the 210 included patients, 24 (11.4%) had ASPECTS between 0 and 5 (Figure 1 and Table 1). Nineteen (9%) patients had ASPECTS between 3 and 5, and 5 (2.4%) between 0 and 2. The distribution of baseline ASPECTS is shown in Supplemental Figure 1. The low ASPECTS group had a higher proportion of women (79% vs 55%, *p* = 0.03) and a higher prevalence of atrial fibrillation (58% vs 36%, *p* = 0.04). Patients with low ASPECTS had higher ischemic core volumes on CTP (median: 71 mL [35–141] vs 5 mL [0–17], *p* < 0.001), worse collateralization on CT angiography (median [IQR]: 1 [0–1] vs 1 [1–2], *p* = 0.01), and less distal occlusions (M2-segment; 13% vs 31%, *p* = 0.02). EVT was less frequently performed in the ASPECTS 0–5 group (25% vs 69%, *p* < 0.001).

**Table 1.** Characteristics of patients with ischemic stroke presenting within 6 and 24 h after stroke onset according to dichotomized ASPECTS.

Variables of interest	Total (*N* = 210)	ASPECTS		*p* Value
		Low (0–5 points) (*N* = 24)	High (6–10 points) (*N* = 186)	
*Clinical characteristics*				
Age (year) – median [IQR]	78 (69–84)	81 (63–86)	78 (69–84)	0.62
Female sex – no. (%)	122 (58)	19 (79)	103 (55)	0.03
Prehospital mRS 0–2 – no. (%)	0 (0–2)	0 (0–2)	0 (0–2)	0.67
Arterial hypertension – no. (%)	145 (69)	15 (63)	130 (70)	0.46
Diabetes mellitus – no. (%)	39 (19)	5 (21)	34 (18)	0.76
Previous ischemic stroke – no. (%)	33 (16)	3 (13)	30 (16)	0.65
Coronary artery disease – no. (%)	39 (19)	4 (17)	35 (19)	0.80
Atrial fibrillation – no. (%)	81 (39)	14 (58)	67 (36)	0.04
*Imaging characteristics* ^a^				
Onset to imaging time (min) – median [IQR]	713 (520–898)	840 (541–1037)	701 (519–854)	0.23
Collateral score – median [IQR]	1 (1–2)	1 (0–1)	1 (1–2)	0.01
CBF < 30% volume (mL) – median [IQR]	8 (0–25)	71 (35–141)	5 (0–17)	<0.001
Mismatch volume (mL) – median [IQR]	69 (39–103)	69 (32–105)	70 (40–102)	0.66
Occlusion site – no. (%)				
Isolated ICA	31 (15)	2 (8)	29 (16)	0.35
ICA	53 (25)	8 (33)	45 (24)	0.33
M1	120 (57)	19 (79)	101 (54)	0.02
M2	60 (29)	3 (13)	57 (31)	0.06
*Treatment characteristics*				
Treatment with intravenous tPA – no. (%)	42 (20)	4 (17)	38 (20)	0.66
Onset to bolus time (min) – median [IQR]	767 (616–954)	867 (721–1344)	758 (608–906)	0.32
EVT – no. (%)	134 (64)	6 (25)	128 (69)	<0.001
Unsuccessful EVT – no. (%)	25 (12)	1 (4)	24 (13)	0.21
Onset to groin puncture time (min) – median [IQR]	896 (683–1076)	1012 (951–1640)	881 (676–1062)	0.05
*Safety characteristics* ^b^				
Symptomatic intracranial hemorrhage – no. (%)	5 (2)	0 (0)	5 (3)	0.42

ASPECTS: Alberta Stroke Program Early CT Score (0–10 points, higher scores indicate less severe early ischemic injury); EVT: endovascular treatment; unsuccessful EVT: modified scale for thrombolysis in cerebral infarction (mTICI) score of <2b; ICA: internal carotid artery; M1/M2: M1/M2-segment of the middle cerebral artery; mRS: modified Rankin Scale (0–6 points, higher scores indicate more severe disability); tPA: intravenous tissue-type plasminogen activator.

a Occlusion sites are not mutually exclusive (if not indicated differently). Mismatch was assessed with automated software (RAPID).

Collateral scores (according to Tan et al.^23^): 0 = absent collaterals, 1 = collaterals filling ⩽50% of the occluded territory, 2 = collaterals filling >50% but <100% of the occluded territory, 3 = collaterals filling 100% of the occluded territory.

b Defined as: occurring within 7 days of acute ischemic stroke and associated with ⩾4 points worsening in NIHSS.

### Association between ASPECTS and ischemic core volumes on automated CT perfusion

In total, 27 (13%) had a large (⩾50 mL) ischemic core on aCTP. We found a strong inverse correlation between ASPECTS and aCTP ischemic core volume (*r* = −0.65, *p* < 0.001; Supplemental Figure 2, Supplemental Table 2) and a weak positive correlation between ASPECTS and collateral status (*r* = 0.25, *p* < 0.001). In patients with very low ASPECTS (0–2 points), median CTP core volume was 158 mL (IQR 74–172 mL), in the ASPECTS 6–10 group 5 mL (IQR 0–17 mL).

### Association between ASPECTS, automated CT perfusion ischemic core volumes, and functional outcome at *3* months

**Figure 2.**
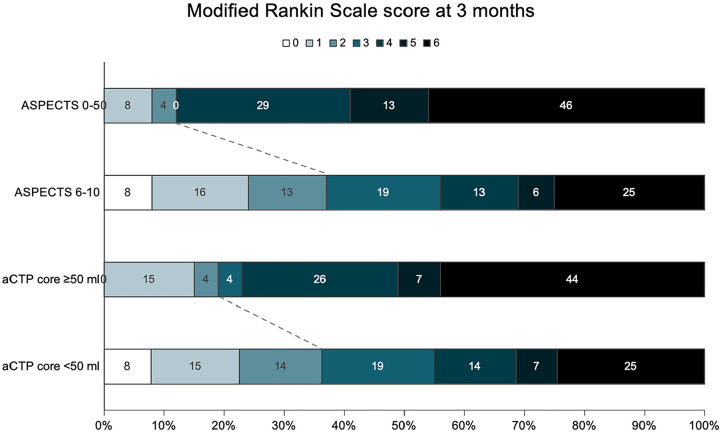
Modified Rankin Scale scores distribution at 3 months according to ASPECTS and automated CT perfusion core volume. The dashed line represents the cutoff between mRS 0-2 (favorable) and 3–6 (unfavorable). ASPECTS: Alberta Stroke Program Early CT Score; aCTP: automated computed tomography perfusion.

**Figure 3.**
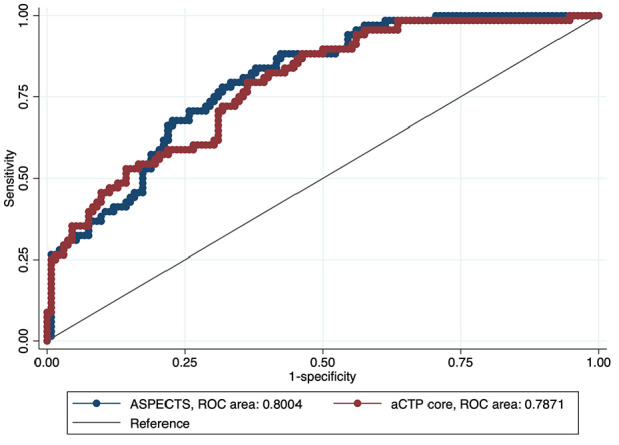
ROC comparison of ASPECTS and automated CTP core-based outcome prediction (favorable outcome at 3 months, defined as a mRS of 0–2). ROC: receiver operating characteristic; ASPECTS: Alberta Stroke Program Early CT Score; aCTP: automated computed tomography perfusion.

At 3 months, 72 (34.3%) patients had favorable outcomes, defined as a mRS of 0–2. Univariable outcome analyses results are presented in Supplemental Table 3. Figure 2 depicts the functional outcome comparisons by baseline CT imaging characteristics (ASPECTS [0–5 vs 6–10], aCTP ischemic core volume [⩾50 mL vs < 50 mL]). ASPECTS and aCTP core volumes were significantly associated with functional outcomes at 3 months. Patients in the ASPECTS 0–5 group had worse functional outcomes compared to the 6–10 group (mRS 0–2: 13% vs 37%, *p* = 0.02; Supplemental Table 4). There was no evidence of multicollinearity, as indicated by variance inflation factor values below 10 (Supplemental Table 5).^13^ Two separate multivariable ordinal regression models with favorable functional outcome as endpoint showed a statistically significant association with, respectively, higher ASPECTS and lower aCTP core volumes (Table 2). In a model including both ASPECTS and aCTP core volumes, only ASPECTS remained associated with functional outcome (ASPECTS: acOR 1.85, 95%CI 1.27–2.70, *p* = 0.001, Table 2). The ROC curve analyses showed comparable diagnostic accuracy in predicting favorable functional outcomes (mRS 0–2) at 3 months (ROC areas: ASPECTS 0.80 [95%CI 0.74–0.86] vs aCTP core 0.79 [95%CI 0.72–0.85]; Figure 3).

**Table 2.** Multivariable analyses of functional outcomes at 3 months according to ASPECTS and automated CT perfusion core volume.

Dichotomized ischemic injury variables^a^		acOR	95%CI	*p* Value
*ASPECTS (high vs low)*				
mRS shift toward lower categories		5.19	2.08 -12.93	0.001
*aCTP core volume (small vs large)*				
mRS shift toward lower categories		4.07	1.63–10.21	0.003
*ASPECTS and aCTP core volume (high vs low; small vs large)*				
mRS shift toward lower categories	ASPECTS	3.55	1.28–9.87	0.02
	aCTP core	2.30	0.84–6.30	0.11
Continuous ischemic injury variables^b^		acOR	95%CI	*p* Value
*ASPECTS (continuous)*				
mRS shift toward lower categories		1.38	1.19–1.60	<0.001
*aCTP core volume (continuous)*				
mRS shift toward lower categories		0.99	0.98–1.00	0.004
*ASPECTS and aCTP core volume (continuous)* ^b^				
mRS shift toward lower categories	ASPECTS	1.85	1.27–2.70	0.001
	aCTP core	0.94	0.63–1.39	0.75

ASPECTS: Alberta Stroke Program Early CT Score (0–10 points, higher scores indicate less severe early ischemic injury); aCTP: automated computed tomography perfusion; mRS: modified Rankin Scale (0–6 points, higher scores indicate more severe disability).

Adjusted common odds ratios (acOR) are reported. We state 95% confidence intervals (CI). Cases with missing data for one or more covariable (*N* = 10) were excluded from the analyses.

Covariables: age, sex, occlusion site (internal carotid artery, M1 or M2 segment of the middle cerebral artery), prior mRS score (i.e., before index event), prior ischemic stroke, intravenous thrombolysis, and performance of EVT.

a High ASPECTS were defined as >5 points. Small CTP core volume was defined as <50 mL.

b Coefficient standardization using z-transformation to account for the different scales of the predictor variables.

## Discussion

In this multicenter cohort of adult aLVO patients admitted between 6 and 24 h after symptom onset, we found that: (i) only a minority of patients presented with signs of a large ischemic core at admission, defined by a visually assessed ASPECTS of 0–5 or an aCTP core volume ⩾50 mL; (ii) higher visually assessed ASPECTS and smaller aCTP ischemic core volumes were associated with favorable 3-month functional outcome in separate models; and (iii) visually assessed ASPECTS were at least comparably accurate to aCTP core volume in predicting favorable functional outcomes.

A pooled analysis^14^ of the SWIFT, SWIFT PRIME, and STAR studies revealed that nearly 89% of patients presenting within 8 h of symptom onset had an ASPECTS of 8–10, suggesting a low early ischemic injury burden. A high baseline ASPECTS was associated with favorable functional outcomes at 3 months. Another study including 224 patients with anterior or posterior LVO showed an ASPECTS >5 in 75% presenting between 6 and 12 h and 77% for those presenting between 12 and 24 h.^15^ These studies suggest that – despite presenting in the late time window – most patients with aLVO have a low to moderate burden of early ischemic injury, possibly related to better leptomeningeal collateralization in high baseline ASPECTS patients. This assumption is supported by the observation that patients in the high ASPECTS group (6–10 points) presented with smaller ischemic core volumes on CTP at baseline.

A moderate correlation between ASPECTS and aCTP ischemic core volumes has been previously described in patients with stroke due to aLVO.^16^ This association was stronger with increasing delay from stroke onset, which could make ASPECTS particularly valuable as a core estimation parameter in the 6–24 h time window.^16^ However, significant variability of aCTP-derived ischemic core volumes was observed within individual ASPECTS subgroups, suggesting that EVT patient selection may be substantially influenced by the imaging selection method.^17^

Recent studies demonstrated that ASPECTS-based patient selection is feasible in patients with extensive ischemic damage on admission,^1–3^ with a possible shift away from the “target-mismatch” imaging concept.^18^ In our study, higher visually assessed ASPECTS and smaller aCTP ischemic core volumes were both associated with favorable 3-month functional outcomes. In direct comparison – incorporating ASPECTS and aCTP ischemic core volumes in the same model – we found that visually assessed ASPECTS was a more reliable predictor of 3-month functional outcome than the RAPID generated aCTP ischemic core volume. Our observations suggest that visually assessed ASPECTS could be at least an equivalent operationalization of early ischemic injury for EVT patient selection in the extended time window than aCTP core volumes derived using automated software.

Our findings may seem contradictory in light of Sui et al.’ recently published results, who demonstrated that within patients treated with EVT, aCTP-defined large core was a better predictor of poor outcome than ASPECTS-defined large core, especially within the early 6 h time window.^6^ The latter finding can be explained by considering the time-dependent correlation between ASPECTS and CT perfusion ischemic core, which gets stronger as time passes. Thus, it seems plausible that aCTP may be preferable to ASPECTS regarding outcome prediction in the early 6 h time window. When comparing our results with those of Sui et al., it should be noted that we focused on the 6–24 h time window, included patients that either received EVT or best medical treatment alone, and the software used for aCTP was different. The latter circumstance must be considered insofar as there are differences in diagnostic accuracy between different software packages.^19^

If our results are reproducible in other cohorts with a larger proportion of patients with greater early ischemic injury, these findings support that the use of visually assessed ASPECTS could substitute the use of automatically generated CTP results for patient selection for EVT in the 6-24 h time window. The advantages of visual ASPECTS evaluation compared to aCTP evaluation include: (i) faster acquisition (due to the lack of post-processing); (ii) lower costs (fees for commercial software for automated evaluation); (iii) lower susceptibility to motion artifacts^5^ with reasonable interrater agreement.^20^ Moreover, CTP maps are prone to core overestimation (“ghost infarct core”^21^) at early presentation and possibly to underestimation (“perfusion scotoma”^22^) at later presentation.

The strengths of our study include: (i) a cohort that is restricted to the Swiss Stroke Registry – a prospectively curated national registry without predefined selection criteria that included patients with both EVT and best medical treatment alone; (ii) the consistent use of a validated and commonly used software for aCTP evaluation (RAPID). Limitations include: (i) lack of randomization with possible residual confounding, especially with regard to changes in EVT selection criteria over time; (ii) limited cohort size, particularly concerning patients with ASPECTS of 0–5; (iii) the retrospective nature of the study with lack of information on which imaging parameter influenced treatment allocation.

## Conclusion

Among adult stroke patients with aLVO presenting 6–24 h after symptom onset, visually assessed ASPECTS demonstrated at least comparable accuracy to automatically generated CTP ischemic core results in predicting favorable functional outcomes at 3 months. This suggests that visual ASPECTS could substitute for aCTP in EVT patient selection within this time window.

## Supplemental Material

sj-docx-1-eso-10.1177_23969873241286691 – Supplemental material for Large ischemic core defined by visually assessed ASPECTS predicts functional outcomes comparably accurate to automated CT perfusion in the 6–24 h window Supplemental material, sj-docx-1-eso-10.1177_23969873241286691 for Large ischemic core defined by visually assessed ASPECTS predicts functional outcomes comparably accurate to automated CT perfusion in the 6–24 h window by Tolga D Dittrich, Anh Nguyen, Peter B Sporns, Anna M Toebak, Lilian F Kriemler, Salome Rudin, Annaelle Zietz, Benjamin Wagner, Filip Barinka, Martin Hänsel, Henrik Gensicke, Raoul Sutter, Christian H Nickel, Mira Katan, Nils Peters, Lars Michels, Zsolt Kulcsár, Grzegorz M Karwacki, Marco Pileggi, Carlo Cereda, Susanne Wegener, Leo H Bonati, Marios Psychogios and Gian Marco De Marchis in European Stroke Journal
