# Supplementary material for: Large ischemic core defined by visually assessed ASPECTS predicts functional outcomes comparably accurate to automated CT perfusion in the 6–24 h window
Source: Eur Stroke J. 2024 Oct 7;10(2):552–9. doi: 10.1177/23969873241286691 (PMC11556663; doi:10.1177/23969873241286691)
Supplement: sj-docx-1-eso-10.1177_23969873241286691 – Supplemental material for Large ischemic core defined by visually assessed ASPECTS predicts functional outcomes comparably accurate to automated CT perfusion in the 6–24 h window [file sj-docx-1-eso-10.1177_23969873241286691.docx]

**Supplementary Material**

**LARGE ISCHEMIC CORE DEFINED BY VISUALLY ASSESSED ASPECTS PREDICTS FUNCTIONAL OUTCOMES COMPARABLY ACCURATE TO AUTOMATED CT PERFUSION IN THE 6-24 HOUR WINDOW**

Dittrich et al.

CONTENTS

[**Supplemental Figure 1.** ASPECTS distribution 2](#_Toc163833009)

[**Supplemental Figure 2.** Association between ASPECTS and automated CT perfusion core volumes 3](#_Toc163833010)

[**Supplemental Table 1.** Data completeness of the covariables of interest 3](#_Toc163833011)

[**Supplemental Table 2.** Pairwise correlations between ASPECTS, collateral status, and automated CT perfusion core volume 4](#_Toc163833012)

[**Supplemental Table 3.** Univariable outcome analyses for a mRS shift toward lower categories at three months 4](#_Toc163833013)

[**Supplemental Table 4.** Comparison of functional outcomes at three months according to ASPECTS and automated CT perfusion core volume 4](#_Toc163833014)

[**Supplemental Table 5.** Multicollinearity diagnostics for the final model using the variance inflation factor 5](#_Toc163833015)

# **Supplemental Figure 1.** ASPECTS distribution


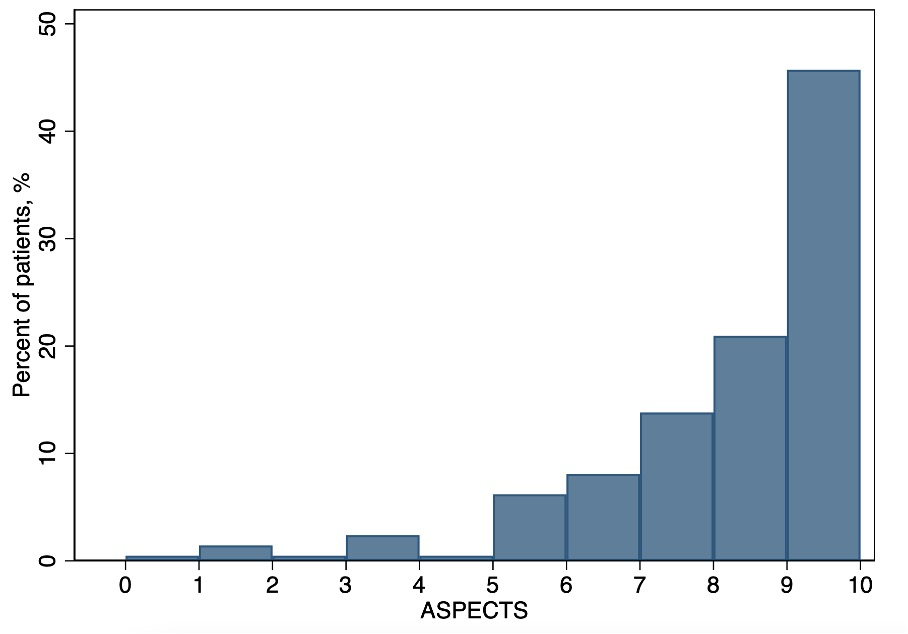


ASPECTS: Alberta Stroke Program Early CT Score

# **Supplemental Figure 2.** Association between ASPECTS and automated CT perfusion core volumes

**
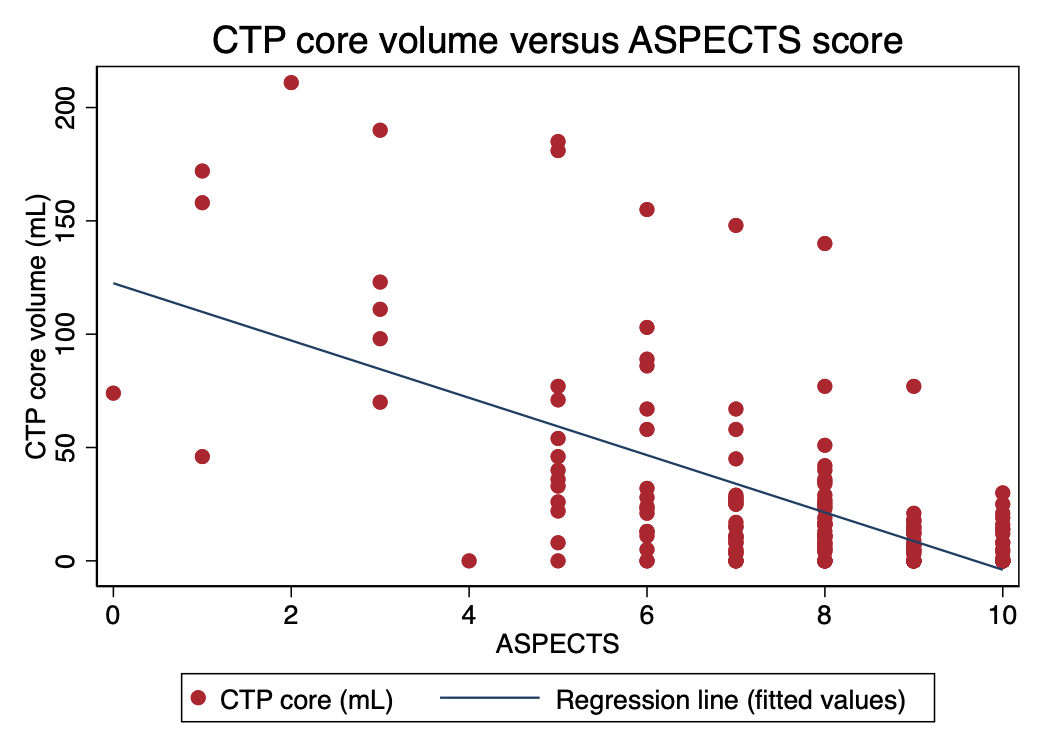
**

# **Supplemental Table 1.** Data completeness of the covariables of interest

|  |  | **Total**  **(N=210)** |
| --- | --- | --- |
| **Clinical characteristics** – no. of missings (%) | | |
| Age |  | 0 (0) |
| Female sex |  | 0 (0) |
| Premorbid mRS |  | 10 (5) |
| Previous ischemic stroke |  | 0 (0) |
| **Imaging characteristics** – no of missings (%)^†^ | | |
| Occlusion site |  | 0 (0) |
| ASPECTS |  | 0 (0) |
| Collateral status |  | 27 (13) |
| CTP core volume |  | 0 (0) |
| **Treatment characteristics** – no of missings (%) | | |
| Treatment with intravenous tPA |  | 0 (0) |
| EVT |  | 0 (0) |
| ASPECTS: Alberta Stroke Program Early CT Score (0-10 points, higher scores indicate less severe early ischemic injury); CTP: computed tomography perfusion; EVT: endovascular treatment; mRS: modified Rankin Scale (0-6 points, higher scores indicate more severe neurological disability); tPA: intravenous tissue-type plasminogen activator.  ^†^ Occlusion sites are not mutually exclusive (if not indicated differently). | | |

# **Supplemental Table 2.** Pairwise correlations between ASPECTS, collateral status, and automated CT perfusion core volume

|  | **ASPECTS** | **Collateral status** | **CTP core volume** |
| --- | --- | --- | --- |
| **Collateral status** | 0.25*** | - |  |
| *N* | 183 |  |  |
| **CTP core volume** | -0.65*** | -0.44*** | - |
| *N* | 210 | 183 |  |
| *** Correlation is significant at the 0.001 level  ASPECTS: Alberta Stroke Program Early CT Score (0-10 points, higher scores indicating less severe early ischemic injury); CTP: computed tomography perfusion.  Collateral status was scored using single-phase CT angiography images (0 points: no collaterals; 1 point: collaterals filling ≤50%; 2 points: collaterals filling >50% but <100%; and 3 points: collaterals filling 100% of the occluded area). | | | |

# **Supplemental Table 3.** Univariable outcome analyses for a mRS shift toward lower categories at three months

|  | | cOR |  | 95% CI |  | p-value |
| --- | --- | --- | --- | --- | --- | --- |
| Age | | 0.95 |  | 0.93-0.97 |  | **<0.001** |
| Female sex | | 0.43 |  | 0.26-0.71 |  | **0.001** |
| Premorbid mRS | | 0.52 |  | 0.41-0.65 |  | **<0.001** |
| Arterial hypertension | | 0.63 |  | 0.38-1.04 |  | 0.07 |
| Diabetes | | 0.81 |  | 0.44-1.50 |  | 0.51 |
| Prior ischemic stroke | | 0.46 |  | 0.23-0.90 |  | **0.02** |
| Coronary heart disease | | 0.87 |  | 0.47-1.59 |  | 0.64 |
| Onset to admission time | | 1.00 |  | 1.00-1.00 |  | 0.37 |
| Occlusion site^†^ | |  |  |  |  |  |
|  | ICA | 1.07 |  | 0.62-1.86 |  | 0.81 |
|  | M1 | 0.47 |  | 0.29-0.77 |  | **0.003** |
|  | M2 | 2.35 |  | 1.38-3.99 |  | **0.002** |
| Treatment with intravenous tPA | | 1.25 |  | 0.68-2.31 |  | 0.48 |
| EVT | | 1.13 |  | 0.69-1.86 |  | 0.63 |
| cOR: common odds ratio; EVT: endovascular treatment; mRS: modified Rankin Scale (0-6 points, higher scores indicate more severe disability; tPA: intravenous tissue-type plasminogen activator.  ^†^ Occlusion sites are not mutually exclusive (if not indicated differently). | | | | | | |

# **Supplemental Table 4.** Comparison of functional outcomes at three months according to ASPECTS and automated CT perfusion core volume

|  |  |  |  |  |  |  | |
| --- | --- | --- | --- | --- | --- | --- | --- |
|  |  | **ASPECTS 0-5**  **(N=24)** |  | **ASPECTS 6-10**  **(N=186)** |  | **P-value** | |
| **Efficacy outcome measure** | |  |  | |  | |  |
|  | mRS 0-2 at 90 days – no. (%) | 3 (13) |  | 69 (37) |  | **0.02** | |
|  |  |  |  |  |  |  | |
|  |  |  |  |  |  |  | |
|  |  | **CTP core ≥50 mL**  **(N=27)** |  | **CTP core <50 mL**  **(N=183)** |  | **P-value** | |
| **Efficacy outcome measure** | |  |  |  |  |  | |
|  | mRS 0-2 at 90 days – no. (%) | 5 (19) |  | 67 (37) |  | 0.06 | |
|  |  |  |  |  |  |  | |
| mRS: modified Rankin Scale (0-6 points, higher scores indicate more severe neurological disability). Chi-square (Χ^2^) test was performed for comparison. | | | | | | |  |

# **Supplemental Table 5.** Multicollinearity diagnostics for the final model using the variance inflation factor

|  | | *VIF* |  | *Tolerance (1/VIF)* |
| --- | --- | --- | --- | --- |
| Age | | 1.22 |  | 0.8184 |
| Female sex | | 1.18 |  | 0.8502 |
| Premorbid mRS | | 1.20 |  | 0.8349 |
| Prior ischemic stroke | | 1.03 |  | 0.9689 |
| ASPECTS | | 1.86 |  | 0.5391 |
| aCTP | | 1.93 |  | 0.5178 |
| Occlusion site^†^ | |  |  |  |
|  | M1 | 1.13 |  | 0.8872 |
|  | M2 | 1.22 |  | 0.8225 |
| Treatment with intravenous tPA | | 1.04 |  | 0.9661 |
| EVT | | 1.15 |  | 0.8733 |
| ASPECTS: Alberta Stroke Program Early CT Score (0-10 points, higher scores indicating less severe early ischemic injury); aCTP: automated computed tomography perfusion; EVT: endovascular treatment; mRS: modified Rankin Scale (0-6 points, higher scores indicate more severe neurological disability); tPA: intravenous tissue-type plasminogen activator; VIF: variance inflation factor.  ^†^ Occlusion sites are not mutually exclusive (if not indicated differently). | | | | |
